# Quality of dying in hospital general wards: a cross-sectional study about the end-of-life care

**DOI:** 10.1186/s12904-021-00862-8

**Published:** 2021-10-12

**Authors:** Filippo Binda, Marco Clari, Gabriella Nicolò, Simone Gambazza, Barbara Sappa, Paola Bosco, Dario Laquintana

**Affiliations:** 1 Department of Healthcare Professions, Fondazione IRCCS Ca’ Granda Ospedale Maggiore Policlinico, Via Francesco Sforza, 35, 20122 Milan, Italy; 2 Department of Public Health and Paediatrics - University of Torino, Via Santena, 5, 10126 Torino, Italy; 3 Department of Healthcare Professions (General Internal Medicine Unit), Fondazione IRCCS Ca’ Granda Ospedale Maggiore Policlinico, Via Francesco Sforza, 35, 20122 Milan, Italy; 4 Department of Healthcare Professions (High-dependency Unit), Fondazione IRCCS Ca’ Granda Ospedale Maggiore Policlinico, Via Francesco Sforza, 35, 20122 Milan, Italy

**Keywords:** End-of-life, Hospital death, Symptom control, Quality of death, Palliative care

## Abstract

**Background:**

In the last decade, access to national palliative care programs have improved, however a large proportion of patients continued to die in hospital, particularly within internal medicine wards.

**Objectives:**

To describe treatments, symptoms and clinical management of adult patients at the end of their life and explore whether these differ according to expectation of death.

**Methods:**

Single-centre cross-sectional study performed in the medical and surgical wards of a large tertiary-level university teaching hospital in the north of Italy. Data on nursing interventions and diagnostic procedure in proximity of death were collected after interviewing the nurse and the physician responsible for the patient. Relationship between nursing treatments delivered and patients’ characteristics, quality of dying and nurses’ expectation about death was summarized by means of multiple correspondence analysis (MCA).

**Results:**

Few treatments were found statistically associated with expectation of death in the 187 patients included. In the last 48 h, routine (70.6%) and biomarkers (41.7%) blood tests were performed, at higher extent on patients whose death was not expected. Many symptoms classified as severe were reported when death was highly expected, except for agitation and respiratory fatigue which were reported when death was moderately expected. A high Norton score and absence of anti-bedsore mattress were associated with unexpected death and poor quality of dying, as summarized by MCA. Quality of dying was perceived as good by nurses when death was moderately and highly expected. Physicians rated more frequently than nurses the quality of dying as *good* or *very good*, respectively 78.6 and 57.8%, denoting a fair agreement between the two professionals (k = 0.24, *P* <  0.001). The palliative care consultant was requested for only two patients.

**Conclusion:**

Staff in medical and surgical wards still deal inadequately with the needs of dying people. Presence of hospital-based specialist palliative care could lead to improvements in the patients’ quality of life.

## Introduction

Dying in an acute hospital is a common occurrence in developed countries [1]. According to a recent study, 45.7% of terminally ill patients, suffering from both oncologic and chronic-degenerative diseases, die in hospital, 44.4% at home, 6.1% in hospice and 3.8% in other settings (residential health facilities, ambulance) [2]. The number of specialized facilities to assist dying patients in Italy has been increasing in the last 10 years, thanks to an explicit legislative act [3], and programs for hospice-type terminal care at home as an alternative to hospitals are growing as well [4]. However, due to administrative issues and chronic shortage of hospice-beds, patients at the end of life (EOL) are often not timely transferred to these facilities, thus continuing to receive therapeutic and diagnostic procedures that have not shown survival benefit [5]. As a consequence, this affects also the quality of care for dying people, reported to be of lower quality in hospital than elsewhere [6]. As long as the hospital continues to be the place where a large proportion of people with terminal illnesses die (especially for patients with neoplastic diseases) [6], a call to keep on improving the EOL care in general medicine wards is required.

Describing treatments and severity of patients’ symptoms in proximity of death would help to identify the main pitfalls and areas for improvement. When death is expected, invasive treatments may be timely suspended, and then healthcare personnel can shift from a curative to a palliative approach. Recognizing this crucial time might determine an overall improvement of EOL care as well.

The efforts that have been made after the abrogation of the Italian law to guarantee access to palliative care service [7] led us to conduct this study, whose aim was to describe how adult patients die in the medical and surgical wards of a large tertiary-level university teaching hospital in the north of Italy. Particularly, we explored how treatments and symptoms severity varied according to expectation of death, as assessed by nurses. Eventually, we investigated the opinion of nurses and physicians about the quality of dying and the clinical management of patients at EOL.

## Methods

### Study design

This single-centre cross-sectional study was carried out in 26 different medical and surgical wards of Fondazione IRCCS Ca′ Granda Ospedale Maggiore Policlinico (Milano, Italy), the largest public research hospital in Italy with 900 beds and more than 36,000 hospitalizations in 2019.

The study enrolled all adult patients (age ≥ 18 years), who died after at least 48 h of hospital stay, between July and December 2019. Patients who died in the emergency room and intensive care units were excluded from the enrolment because the type of care provided there is strictly aimed at keeping patients alive with different end-of-life management.

Data were prospectively collected by an independent nurse using a case report form created for the study, including patients’ demographic and clinical characteristics. Information about room setting and contents of EOL communication with relatives or caregivers were collected as well from the same source. By 48 h from patient’s death, the same independent nurse interviewed the physician and nurse responsible for taking care of the patient at the time of death. Medical and nursing records were assessed for data quality purposes in order to check for inconsistencies between these records and the electronic database used for the analysis. Deaths were classified as *highly*, *moderately*, or *not expected* on the basis of a question asked to the nurses and physicians in charge: “Had somebody told you yesterday that the patient would have died within a day, how far would have you agreed?”. Also, nurses and physicians were asked to rate the quality of dying on a 5-point Likert scale from *very poor* to *very good*. Nurses were also asked to classify the intensity of reported symptoms as *severe*, *moderate* or *absent*, whenever these could be assessed.

In the present study, Norton’s scale was used as an assessment tool to predict the risk of suffering from pressure ulcers, as routinely implemented by nurses within our hospital [8]. The scale takes into consideration 5 domains: physical condition, mental state, physical activity, mobility and incontinence. Indicatively, a Norton rating below 9 means very high risk, 10 to 13 means high risk, 14 to 17 medium risk and above 18 means low risk. The level of consciousness was assessed by the mean of the Ramsay Sedation Scale, which divides a patient’s level of sedation into six categories ranging from severe agitation to deep coma [9, 10].

Compared to previous findings [11], at least 159 individuals were needed to detect 10% change in the proportion of *good/very good* dying quality with 90% power using 5%-level two-sided test.

The local Ethics Committee approved the study protocol (ethics approval number 1099_2019). Data were collected and stored following the provisions of the Italian Data Protection Authority regarding personal data security and informed consent was obtained from all nurses and physicians participating in the study.

### Data analysis

Metrics were reported as mean and standard deviation (sd) or counts and percentage (%). Statistical association between type of treatments (presence versus absence) or symptoms (severe versus moderate or absent*)* and death as expected by nurses was explored using Chi-square statistics or Fisher exact test, as appropriate. Only nurses’ judgment was used to stratify these analyses, due to other studies showing lower accuracy to predicting death by physicians compared to nurses [12, 13]. Throughout summary tables, proportions are presented with 95% confidence interval (CI).

In order to explore the association between quality of dying (*very poor* through *very good*), nurses’ expectation about death (*not expected*, *moderately* and *highly expected*), nursing treatments delivered (anti-bedsore mattress positioning, body and oral hygiene care, artificial tears administration, tracheal suctioning, active mobilization, vascular access management) and patients’ characteristics (sex, age and scoring at Norton’s scale), we selected a multivariate approach using multiple correspondence analysis (MCA) [14]. MCA takes multiple categorial variables and seeks to identify association between them. Like other multivariate methods, MCA is also a dimension reducing technique, therefore it represents data as points in 2-dimensional space (biplot). The results can be visualized through the biplot; as variables become more similar, the closer they are grouped together.

With regard to the agreement between nurses and physicians about quality of dying, we used Cohen’s kappa (k), and we reported the 95%CI as well for the k coefficient. For all analyses, *P*-values were two-sided, and *P* <  0.05 was considered to be statistically significant. All the analyses were performed using R Core Team, version 3.6.2, with package *factoMiner* and *factoextra* added.

## Results

Data were collected on 187 out of 224 patients deceased during the study period (37 patients were excluded because their death occurred < 48 h after admission to the ward). The patients’ main characteristics are reported in Table 1. The mean(sd) age was 78.6(12.8) years with an average occurrence of death after 13.9(11.2) days from admission.

**Table 1:** General characteristics of patients (*n* = 187)

**Variable**	**N (%)**
Age group, *years*	
< 64	26 (13.9)
64–74	35 (18.7)
75–84	52 (27.8)
> 85	74 (39.6)
Males	105 (56.1)
Females	82 (43.9)
***Hospital admission***	
Emergency admission	157 (83.9)
Planned admission	30 (16.1)
***Type of hospital ward***	
Medical ward	169 (90.4)
Surgical ward	18 (9.6)
***Primary diagnosis***	
Respiratory	63 (33.7)
Oncologic	42 (22.5)
Cardiovascular	23 (12.3)
Infectious	21 (11.2)
Neurologic	18 (9.6)
Gastrointestinal	11 (5.9)
Nephrologic	8 (4.3)
Other diagnosis	1 (0.5)
***Time of death***	
Daytime hours (7 AM – 9 PM)	118 (63.1)
Nighttime hours (9 PM – 7 AM)	69 (36.9)
***Cause of death***	
Cardiorespiratory arrest	126 (67.4)
Sepsis / Multiple organ failure	44 (23.5)
Liver failure	10 (5.4)
Hemorrhage	7 (3.7)

According to the nurses’ clinical judgment, death was *highly expected* in 54.4% (102/187) of patients, whereas it was *moderately expected* and *not expected* for the 33.6% (61/187) and 12.8% (24/187) of cases, respectively. Few treatments were found statistically associated with expectation of death, as described in Table 2. In the last 48 h, both routine (132/187, 70.6%) and biomarkers (78/187, 41.7%) blood tests were performed, at higher extent on patients whose death was *not expected*. Chest radiological examinations (37/187, 19.8%) were statistically associated with nurses’ judgment, and these procedures were mostly prescribed to the group of patients whose death was *not expected* either (*P* = 0.007). At the time of death, in the 89.3% (167/187) of the sample, emergency procedures were not performed. Overall, emergency procedures showed statistically significant difference between expectation of death as assessed by nurses (*P* <  0.001), and all the listed procedures had a low occurrence in the group whose death was *highly expected*. It is worth noting that the medical emergency team was activated to perform cardiopulmonary resuscitation on five patients only.

**Table 2:** Treatments and procedures in the last 48 h of life, stratified by nurses’ judgement on expectation of death

**Treatments and procedures**	**Death not expected (*n* = 24)**		**Death moderately expected (*n* = 61)**		**Death highly expected (*n* = 102)**		***P*-value**
	***n* (%)**	**95% CI**	***n* (%)**	**95% CI**	***n* (%)**	**95% CI**	
Routine blood tests	23 (95.8)	78.8–99.8	45 (73.8)	60.9–84.2	64 (62.7)	52.6–72.1	**0.002**
Biomarkers blood tests	16 (66.7)	44.7–84.3	26 (42.6)	30.0–55.9	36 (35.3)	26.1–45.4	**0.019**
Chest radiography	10 (41.7)	22.1–63.4	13 (21.3)	11.8–33.6	14 (13.7)	7.6–21.9	**0.007**
CT scan	6 (25.0)	9.8–46.7	7 (11.5)	4.8–22.3	14 (13.7)	7.6–21.9	0.267
Intravenous fluid therapy (> 500 ml)	17 (70.8)	48.9–87.4	48 (78.5)	66.1–87.9	78 (76.5)	67.1–84.3	0.744
Blood transfusion	6 (25.0)	9.8–46.7	12 (19.7)	10.6–31.9	10 (9.8)	4.8–17.3	0.078
Hemodialysis	3 (12.5)	2.6–32.4	2 (3.3)	0.4–11.4	5 (4.9)	1.6–11.1	0.207
Low flow oxygen therapy	20 (83.3)	62.6–95.2	56 (91.8)	81.9–97.3	89 (87.3)	79.2–93.1	0.450
Non-invasive ventilation	4 (16.7)	4.7–37.4	12 (19.7)	10.6–31.9	13 (12.7)	6.9–20.7	0.490
Artificial nutrition	5 (20.8)	4.6–37.1	17 (27.9)	16.6–39.1	41 (39.0)	30.7–49.7	0.098
Urinary catheter	14 (58.3)	36.6–77.8	40 (65.6)	52.3–77.3	69 (67.6)	57.6–76.5	0.687
Enema for bowel evacuation	6 (25.0)	9.8–46.7	9 (14.8)	7.0–26.2	6 (5.9)	2.2–12.4	**0.016**
***Emergency procedures***							
Cardiopulmonary resuscitation	4 (16.7)	4.7–37.4	3 (4.9)	1.0–13.7	2 (1.9)	0.2–6.8	**0.010**
Pulmonary ventilation	5 (20.8)	7.1–42.1	6 (9.8)	3.7–20.1	4 (3.9)	1.1–9.7	**0.019**
Vasopressors drugs	7 (29.2)	12.6–51.1	3 (4.9)	1.0–13.7	1 (0.9)	0.02–5.2	**< 0.001**
No emergency procedures	16 (66.7)	44.7–84.3	54 (88.5)	77.7–95.2	97 (95.1)	88.9–98.4	**< 0.001**

*Abbreviations*: *95% CI* 95% confidence interval, *CT scan* Computed tomography scan

The level of consciousness, encoded with Ramsay Sedation Scale, was severely compromised (from sluggish response to a light glabellar tap or loud auditory stimulus to unresponsive to external stimuli, including pain) in 49.0% (50/102) of patients whose death was *highly expected.* By the contrary, 79.2% (19/24) of those whose death was *not expected* were awake (i.e., oriented and quiet or anxious and restless). Analgesic drugs (i.e., opioids and nonsteroidal anti-inflammatory drugs) were administered in the 76.7% (99/129) of patients with pain. All patients with primary oncologic disease received opioids. The palliative care consultant was requested for only two patients (1.1%), and the intensivist (25/187, 13.4%) and the infectivologist (17/187, 9.1%) were the most requested consultants.

As summarized in Table 3, severity of symptoms shows evidence of association with expectation of death, as assessed by nurse, with regard to respiratory fatigue only (*P* = 0.0276). However, the majority of symptoms classified as severe were reported when death was highly expected.

**Table 3:** Severe signs and symptoms stratified by expectation of death, as perceived by nurses

**Signs and symptoms**	**Patients with severe symptoms**	**Death not expected**		**Death moderately expected**		**Death highly expected**		***P*-value**
	***n* (%)**	***n* (%)**	**95% CI**	***n* (%)**	**95% CI**	***n* (%)**	**95% CI**	
Asthenia^a^	85/106 (80.2)	13 (15.3)	8.4–24.7	32 (37.6)	27.3–48.7	40 (47.1)	36.1–58.2	0.120
Pain	47/129 (13.6)	10 (21.3)	10.7–35.6	13 (27.7)	15.6–42.6	24 (51.1)	36.1–65.9	0.129
Urinary incontinence	96/124 (77.4)	11 (11.5)	67.7–85.3	29 (30.2)	21.2–40.4	56 (58.3)	47.8–68.3	0.559
Respiratory fatigue	59/121 (51.2)	8 (13.5)	6.1–24.5	28 (47.5)	34.6–60.5	23 (38.9)	26.5–52.5	**0.028**
Confusion^a^	28/77 (36.4)	5 (17.9)	6.0–36.9	9 (32.1)	15.8–52.3	14 (50.0)	30.6–69.3	0.718
Agitation^a^	32/77 (41.6)	8 (25.0)	11.4–43.4	14 (43.7)	26.3–62.9	10 (31.3)	16.2–50.1	0.271
Gasping respiration	45/112 (40.2)	3 (6.7)	1.4–18.3	11 (24.4)	12.8–39.4	31 (68.9)	53.3–81.4	0.085
Fecal incontinence	55/96 (57.3)	3 (5.4)	1.1–15.0	20 (36.4)	23.8–50.4	32 (58.2)	44.1–71.3	0.144
Bronchial secretions	41/94 (43.6)	2 (4.9)	0.6–16.5	13 (31.7)	18.1–48.1	26 (63.4)	46.9–77.8	0.186
Pressure ulcers	39/92 (42.4)	4 (10.3)	2.9–24.3	11 (28.2)	15.0–44.8	24 (61.5)	44.5–76.6	0.609
Cough	9/52 (17.3)	1 (11.1)	0.3–48.2	3 (33.3)	7.4–70.0	5 (55.6)	21.2–86.3	1.0
Fever	31/51 (60.8)	7 (22.6)	9.6–41.1	6 (19.3)	7.4–37.4	18 (58.1)	39.1–75.4	0.089
Nausea / vomiting	20/34 (58.8)	2 (10.0)	1.2–31.7	8 (40.0)	19.1–63.9	10 (50.0)	27.2–72.8	0.095

*Abbreviation*: *95% CI* 95% confidence interval

^a^Percentages calculated excluding 67 patients with severe impaired consciousness

**Fig. 1:**
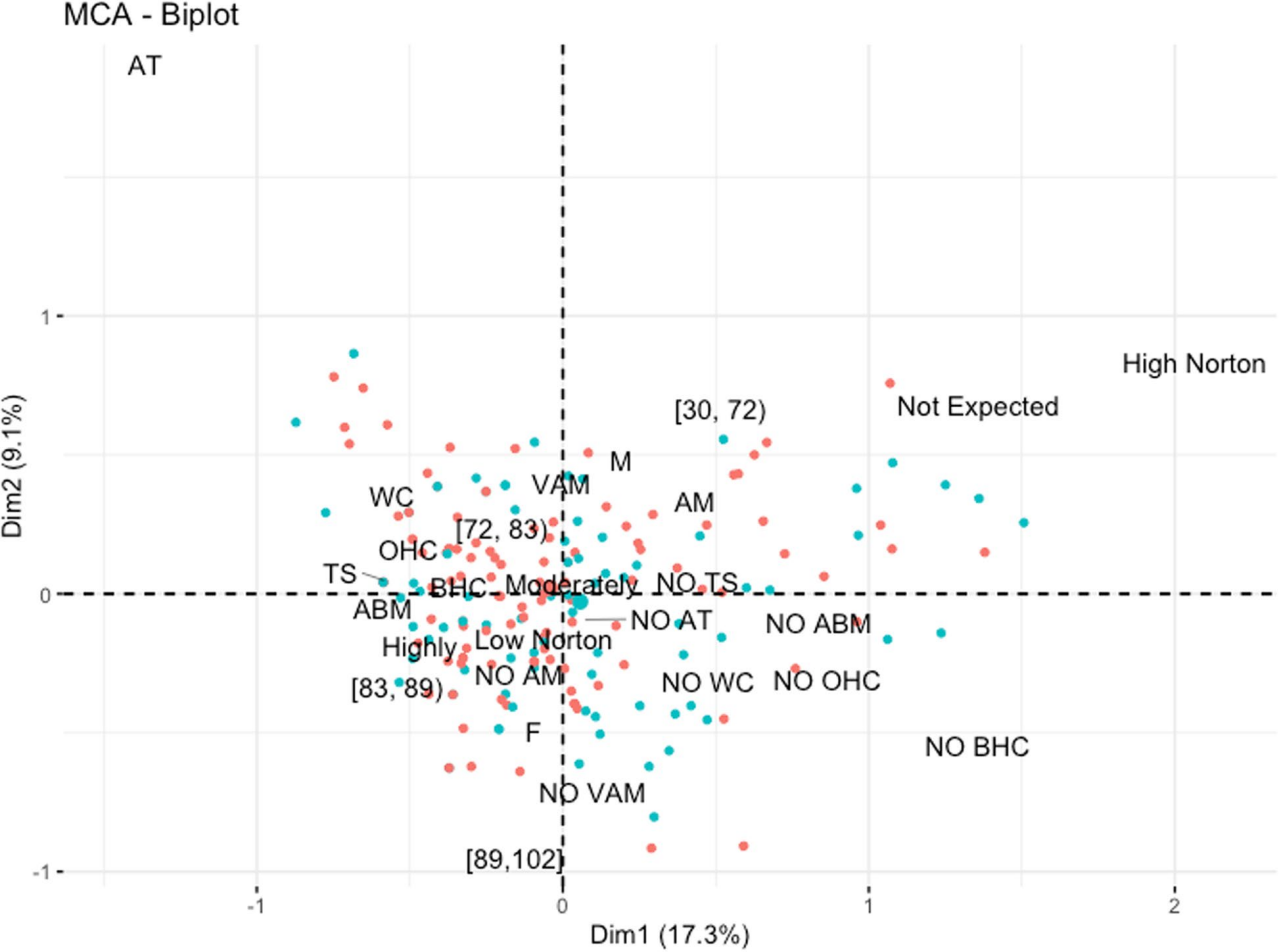
Biplot containing individuals (dots) and variables categories in two MCA dimensions. Blue filled dots denote individuals with very poor/poor/average quality of death whereas red filled dots patients with good or very good quality of death. MCA referred to nurses’ expectation about death (Not Expected, Moderately and Highly Expected) and nursing care interventions delivered or not (NO) to males (M) and females (F): anti-bedsore mattress (ABM; NO ABM), body hygiene care (BHC; NO BHC), oral hygiene care (OHC; NO OHC), artificial tears (AT; NO AT), tracheal suctioning (TS; NO TS), active mobilization (AM; NO AM) and vascular access management (VAM; NO VAM). Norton’s score is reported as “Low Norton” (≤ 12) and “High Norton” (≥ 13). Age is reported as quartiles: [30,72), [72, 83), [83,89), [89,102], where “(“…“)” and “[“… “]” denotes open and closed intervals. The Dim1 axis (i.e. “Pressure ulcer management”) is the first dimension along which the sample show the largest variation, whereas Dim2 (i.e., “Comfort”) is the second most important dimension, and it is orthogonal to the Dim1, which explain the 8.9% of variation in the data

Nursing care was provided until the moment of death. Hygiene of the body (partial or total) and oral care, together with the change of bedding, were the most frequent interventions provided by nurses. The relationship among these activities and nurses’ clinical judgment about expectation of death and quality of dying is summarized in Fig. 1. Two MCA dimensions – termed “pressure ulcer management” and “comfort care” – were identified. For the first, a high Norton score and absence of anti-bedsore mattress were associated with death as not expected and poor quality of dying. By contrary, age above 83 years, lack of active mobilization, female sex and administration of artificial tears appeared to cluster with death as highly expected and with a good quality of dying. On the whole, the MCA biplot shows a global pattern along the first dimension for patients whose death was qualitatively assessed as *good or very good,* mostly stretched on the left side of the plot. When death was *moderately* and *highly expected*, quality of dying was perceived as good by nurses, and it was associated with several nursing interventions. All these variables were the most important in explaining the variability of the whole sample.

Table 4 reports the clinical judgment of nurses and physicians about EOL quality. The Cohen’s kappa statistics showed fair agreement between the two professionals (k = 0.24, 95%CI 0.14 to 0.34, *P* <  0.001). In particular, physicians rated more frequently than nurses the quality of dying as *good*/*very good,* respectively 78.6 and 57.8%. Agreement between physicians and nurses about expectation of death was slightly better (k = 0.32, 95%CI 0.20 to 0.44, *P* < 0.001): 54.5 and 66.3% rated death as *highly expected*, respectively.

**Table 4:** Nurses and physicians’ opinion on the quality of the end of life

**End of life quality**	**Nurses**		**Physicians**	
	***n* (%)**	**95% CI**	***n* (%)**	**95% CI**
Very good	14 (7.5)	4.2–12.3	35 (18.7)	13.4–25.0
Good	94 (50.3)	42.9–57.6	112 (59.9)	52.5–66.9
Average	47 (25.1)	19.1–31.9	30 (16.0)	11.0–22.0
Poor	23 (12.3)	7.9–17.8	7 (3.7)	1.4–7.5
Very poor	9 (4.8)	2.2–8.9	3 (1.6)	0.3–4.6

*Abbreviation*: *95% CI* 95% confidence interval

The analysis of the room setting found that 20.9% (39/187) of patients died in a single room whereas the majority (79.1%, 148/187) in multiple occupancy rooms (two to three beds). However, intimacy was always guaranteed for those who died in multiple rooms. Family members or caregivers had no visit restrictions in the 48 h before death and they were physically present at the patient’s bedside in 69.6% (71/102) of cases whose death was *highly expected* compared to 2/24 (8.3%) *not expected*. The communication with patient’s relatives or caregivers regarding the extreme severity of clinical conditions was carried out in 81.8% (153/187) of cases. Willingness to organs and tissues donation was recorded in 9.8% of patients (15/153), resulting in the corneal tissue removal procedure only.

## Discussion

This study analysed treatments, symptoms and clinical management of adult inpatients at the end of their life. Notably, the majority of patients died in internal medicine wards where patients presenting with exacerbations of their chronic diseases are frequently hospitalized [15]. Except for a few procedures which were reasonable to perform in those patients whose death was not expected, the presence of clinical activities such as CT scanning, blood transfusions and artificial nutrition in the 48 h before death suggests the persistence of an attitude that can lead to overtreatment. Quality of dying as judged by nurses was rated good for nearly 6 patients over 10, which can be considered yet unsatisfactory, compared to the previous Italian study [11].

Routine blood tests were one of the major diagnostic procedure performed in patients whose death was *highly expected*. Many patients underwent a considerable volume of blood samples for laboratory tests during the last hours of life [16], phenomenon already described in previous studies, where at least 50% of patients whose death was highly expected performed blood tests before dying [11, 17]. Literature widely describes that also radiological procedures for diagnostic purposes are frequently performed in patients with poor prognosis admitted to hospice [18], even if in the present study the radiological exams (especially chest radiography) have been performed mainly in patients whose death was *not expected*. Particularly, the uncertainty regarding disease trajectory and prognosis has frequently been cited by clinicians as an excuse to postpone EOL care discussions, thus moving forward diagnostic path and treatments [19].

The intensivist was the most requested consultant, and the main responsibility was to contribute to the *quoad vitam* prognostication. This could explain the very small number of activations of the medical emergency team to perform cardiopulmonary resuscitation. Thus, the main activity of the intensivist was contributing to the decisions making over the appropriate intensity of care, both for patients suffering from end-stage chronic-degenerative and neoplastic diseases, as well as for patients with acute clinical conditions and poor prognosis [20, 21]. However, not all intensivists can make decisions about the appropriateness of treatments (i.e., administration of new antibiotics) or about the suitability of escalation to high dependency or intensive care. This is also acknowledged by the Italian Society of Anaesthesia, Analgesia, Resuscitation and Intensive care (SIAARTI), which has published specific recommendations to guide EOL decision-making for patients outside the intensive care unit [22].

Performing diagnostic and therapeutic procedures clashes with the problem of symptoms control in the last hours of patients’ life. In a large proportion of patients, the level of consciousness was severely compromised, thus they were not able to communicate appropriately the characteristics of pain. For this reason, it is recommended using clinical scoring tools for pain evaluation which may include patients’ behavior and not only professionals’ clinical judgment [23]. Nevertheless, reducing *peri-mortem* to a complex and demanding measuring act may be an obstacle for the final decision to care and not to cure.

Amidst symptoms, an effective pain control management is expected in most patients, and physicians should be comfortable in prescribing repeatable dose of analgesic drugs to reach the analgesic peak effect [24]. In this study, all patients with cancer received opioids; as documented in the last national report [25], their usage seems to be constantly increasing. However, despite the administration of analgesic drugs, pain control was not always achieved and different studies show that an important percentage of patients without cancer (25–40%) does not receive an adequate pain-relieving treatment [26]. In addition to pain, the presence of respiratory symptoms (i.e., dyspnea), which mainly characterized the present cohort, worth a further reflection. Together with agitation, severe respiratory fatigue was associated with death *moderately expected*, as it was a red flag along the nursing perception of the overall deterioration of clinical conditions. These findings may be of interest of the consultant in palliative medicine [27], whose competencies were requested in a small number of patients, just because the hospital does not have a palliative care service.

As regards nursing care, patients continued to receive body hygiene, active mobilization and vascular access management until the moment of death. Patients with a lower Norton score were at high risk of developing pressure ulcers, and this might explain the great number of nursing interventions received, mostly related to skin care and prevention of pressure ulcers onset, such as anti-bedsore mattress positioning [28]. Body and oral hygiene care are very important nursing interventions to provide comfort to the bedridden patients [29]. In particular, poor oral hygiene is the most common cause of mouth problem [30], especially in weakened and fatigued patients. Nurses perceived a good EOL quality when they carried out a high number of interventions to old and frail patients, as summarised by MCA. Despite different cultural aspects could contribute to the concept of *good death*, dying without discomfort and suffering is considered a good way of dying in any culture [31]. In order to improve the quality of EOL phase, treatments and nursing interventions are not always enough to relieve symptoms but it is also necessary to timely involving the family to support patients emotionally [32]. On the whole, the present findings show how interventions to ensure comfort and intimacy for patients whose death was *highly expected* were frequently carried out. Correct information regarding the severity of clinical conditions and the presence of family members, without any time constraints, were ensured to almost the totality of patients. These aspects were also included in the *Liverpool Care Pathway* model, a protocol that has been adopted in the United Kingdom for a decade, which help physicians and nurses to increase the quality of treatments in the EOL care [33].

The clinical judgment of nurses and physicians showed a moderate agreement about expectation of death. This is not surprising, considering that identification of the EOL phase is incredibly difficult for the healthcare staff. Defining when a patient is in a phase of stability or instability, worsening or in a terminal phase (phase illness) can be very challenging [34], particularly in patients without cancer [35]. However, prognostic information remains necessary not only for patients and their families, but also for healthcare providers, in order to guide their action and offer necessary interventions. Unfortunately, survival predictions made by clinicians suffer from their subjectivity and sometimes are overly optimistic and not always reliable [36, 37]. Given the time spent at patients’ bedside, nurses could be the first to recognize patients’ end of life, potentially making a significant contribution to the EOL quality [38]. In fact, nurses and physicians showed also different rates of agreement about the quality of EOL in the present study, suggesting that they may share with physicians the same reflection about expectation of death, ultimately different in terms of perceived quality. However, nurses, whose training is more patient-oriented, mostly perceived the overall care as *good* when they performed many interventions, as commented in 2005 [11] and confirmed as well by our findings using MCA. This might constitute a kind of illusory correlation that links quality to the amount of delivered care, which deserves further attention.

Divergencies between nurses and physicians can have various origins. First, training on EOL issues is very inhomogeneous within professionals and between different professional profiles, and the lack of competencies is recognized as the most important barrier to begin EOL discussion [39]. Underestimating the need for information [40] and the fear of taking away the patient’s hope are two crucial aspects that may negatively influence healthcare professionals towards EOL discussion [41]. These could be directly linked to the general theme of patients’ empowerment through shared decision making, by which patients and clinicians work together to make optimal decisions that align with what matters most to patients. The bottom line is that healthcare professionals do not receive enough training or mentoring during their academic years to recognize when it is time to switch from cure to comfort. This kind of gap might favour disagreement within the multidisciplinary team as well, with nurses experiencing some distress when they disagree with the appropriateness of the medical treatments [42, 43]. On the whole, the different professional background and professional aims may be responsible for the differences encountered in the present study.

### Study limits

The study has several limitations. The sampling was limited to our Institution and generalization should be made with cautious to smaller Italian hospitals. The expectation of death was asked only after the patient had died, and the subjectivity of the answers provided by nurses and physicians during the interviews could have overestimated the quality of EOL management. This is an unavoidable limitation for assessment of the dying experience. However, the limited time-window used to assess quality of dying in the present study should have minimized recall bias. Future studies should benefit from combining surprise question and clinical predictors to formulate short-term prognosis of patients dying in medical and surgical wards [44, 45].

## Conclusion

Overall, our general hospital is still not adequate to meet the needs of dying people. The presence of hospital-based specialist palliative care could lead to improvements in the patients’ quality of life; however, the reduced number of palliative care consultations and the poor symptoms control suggest that the bottom-line issues with EOL are not only related to hospital organization but also to intrinsic factor of each healthcare profession.

## Data Availability

The datasets generated during and/or analyzed during the current study are not publicly available but are available from the corresponding author on reasonable request.

## Abbreviations

CT: Computed tomography
EOL: End of life
MCA: Multiple correspondence analysis
SIAARTI: Società Italiana Anestesia, Analgesia, Rianimazione e Terapia Intensiva
